# A Tumor Profile in Primary Immune Deficiencies Challenges the Cancer Immune Surveillance Concept

**DOI:** 10.3389/fimmu.2018.01149

**Published:** 2018-05-24

**Authors:** Daniel Satgé

**Affiliations:** Institut Universitaire de Recherche Clinique, Biostatistics, Epidemiology and Public Health, Team Cancer EA 2415 and Oncodéfi, Montpellier, France

**Keywords:** primary immune deficiencies, immune surveillance, cancer incidence, tumor profile, cancer, lymphoma, gastrointestinal cancer, virus-induced cancer

## Abstract

Under the concept of cancer immune surveillance, individuals with primary immune deficiencies would be expected to develop many more malignancies and show an excess of all types of cancers, compared to people with a normal immune system. A review of the nine most frequent and best-documented human conditions with primary immune deficiency reveals a 1.6- to 2.3-fold global increase of cancer in the largest epidemiological studies. However, the spectrum of cancer types with higher frequencies is narrow, limited mainly to lymphoma, digestive tract cancers, and virus-induced cancers. Increased lymphoma is also reported in animal models of immune deficiency. Overstimulation of leukocytes, chronic inflammation, and viruses explain this tumor profile. This raises the question of cancers being foreign organisms or tissues. Organisms, such as bacteria, viruses, and parasites as well as non-compatible grafts are seen as foreign (non-self) and identified and destroyed or rejected by the body (self). As cancer cells rarely show strong (and unique) surface antibodies, their recognition and elimination by the immune system is theoretically questionable, challenging the immune surveillance concept. In the neonatal period, the immune system is weak, but spontaneous regression and good outcomes occur for some cancers, suggesting that non-immune factors are effective in controlling cancer. The idea of cancer as a group of cells that must be destroyed and eliminated appears instead as a legacy of methods and paradigms in microbiological medicine. As an alternative approach, cancer cells could be considered part of the body and could be controlled by an embryonic and neonatal environment.

## Introduction

According to the concept of immune surveillance proposed by Burnet (1), the immune system identifies and destroys nascent cancers (2, 3). This model is based on the idea that cancers, which are produced by an organism’s cells, present specific antigens recognized by the immune system. Accordingly, compared to normal individuals, individuals with weakened or deficient immune systems should develop an excess of all types of cancers, since white blood cells in immune-compromised patients are less able to recognize and destroy malignant cells.

In this study, the concept of immune surveillance was examined in nine of the most well-known and well-characterized primary immune deficiencies (PIDs) chosen from 300 conditions each with a simple gene defect in the context of immune impairment (4). The immune surveillance concept predicts a global increase in cancer in these PIDs, but instead these conditions associated with a narrow spectrum of particular malignancies. This suggests that several immune processes, such as overstimulation of immune cells, viral infections, reactions to impaired bone marrow tissue, and chronic inflammation, influence cancer in PIDs. The particular distribution of cancers in primary immune-deficient humans and in murine models challenges the concept of immune surveillance as the major cause of increased cancer frequency in these diseases. This observation raises the question of the strategic place of immune mechanisms in diseases that do not arise from intrinsically foreign tissue.

## Materials and Methods

Nine PIDs were selected from 300 immune deficiency diseases with single gene defects (4), on the basis of their frequency and sufficiently documented cancer occurrences. Less frequent conditions were included if significantly associated with cancers. The diseases were chosen from the most frequently cited conditions in recent cancer and PID epidemiological studies (4–7) and from the most frequently cited conditions in PID and cancer reviews (8–11).

We excluded diseases deemed not well defined for a sufficiently long period (preventing assessment of a number of people with precisely the same condition) and diseases with a chromosome breakage-DNA repair defect (to prevent bias due to their effects on oncogenesis). The following conditions were retained, selective IgA deficiency, common variable immune deficiency (CVID), X-linked agammaglobulinemia, X-linked hyper-IgM syndrome, cartilage-hair hypoplasia, Warts,-hypogammaglobulinemia,-infections, and myelokathexis (WHIM) syndrome, severe congenital neutropenia, and natural killer (NK) cell deficiency. For each condition, a search in PubMed was conducted, cross-searching the name of the disease with the words “cancer” and “malignancies.” Articles were selected if their title indicated information on health problems, cancer occurrence, and cancer frequency, or the functional or structural impact of the disease on tissues. Articles reporting *in vitro* experimental data were excluded.

## Results

From the 1,112 identified articles, 223 abstracts were selected for reading, 152 articles were read in full, and 80 articles and book chapters were included in the bibliography.

Table 1 displays cancer distributions in the nine chosen PID conditions (4–6, 9, 10, 12–44).

**Table 1:** Frequency of nine inherited diseases with primary immune deficiency and their cancer risk.

Diseases	Disease frequency	Global cancer risk (fold increase) and/or prevalence (%)	Over-represented cancers	Comments
Selective IgA deficiency (5, 12–15)	1/143–1/965	×1.31	Gastric cancers (×1.64–5.4) Lymphoma × (1.68–2.6)	Lung cancer decreased

CVID (4–6, 16, 17)	1/10,000–100,000	×1.19–3	Lymphoma (×8.4–18.6) Gastric carcinoma (×5–16.2)	No virus-related cancers^a^ Decrease frequency of these cancers usual in children and adults

X-linked agammaglobulinemia (10, 18–21)	1/200,000	Increased	Gastric carcinoma Intestinal carcinoma	Chronic infections *Helicobacter pylori*

X-linked hyper-IgM syndrome (22–25)	1/1,000,000	Increased	Liver carcinoma Gallbladder carcinoma Pancreas carcinoma	Frequent cholecystitis Hepatitis

Wiskott–Aldrich syndrome (26–29)	1/10,000–10,000	Increased	Lymphoma CNS lymphoma	Virus-related cancers (EBV infections)

Cartilage-hair hypoplasia (30–33)	NA^b^	×7 45% at 65 years	Lymphoma (×90) Skin cancer (×33)	

WHIM syndrome (9, 34, 37)	1/4,000,000	30% at 40 years	Lymphoma Genital and ENT squamous cell carcinoma	Viral infections

Severe congenital neutropenia (38–40)	1/100,000	31%	Acute myeloid leukemia (AML) and myelodysplastic syndrome	Bone marrow failure G-CSF treatment

Natural killer cell deficiency (41, 44)	NA	Increased	Acute myeloid leukemia, carcinoma of genital organs and skin cancer, and breast carcinoma	Myelodysplasia, HPV, and EBV infections, GATA2 interacts with GATA3^c^

*NA, not available; EBV, Epstein–Barr virus; HPV, human papilloma virus; CVID, common variable immune deficiency; ENT, ear nose and throat*.

*^a^EBV, HPV*.

*^b^1/1, 000–2,000 in Amish people in USA and 1/23,000 in Finland, very rare in other countries*.

*^c^GATA 3 interacts with GATA 2 which is involved in the growth control of breast cancer*.

### Cancer Frequency in PIDs

Based on the concept of immune surveillance, people with PIDs who are less able to eliminate cancer at its early stages should develop an excess of malignancies, particularly an excess of the most frequent cancers, leukemia, brain tumors, lymphomas, embryonic tumors, and germ cell tumors in childhood and colon, breast, lung, and prostate cancers in adults. The nine PIDs chosen for this review, based on their frequency and documented cancer occurrence, account for a large proportion of all PID patients. For instance, seven of the nine diseases per the United States Immune Deficiency NETwork accounted for 56% (2,047/3,658) of enrolled conditions (4). These diseases are also the most frequent conditions cited in reviews on PID (8, 10, 11), if we exclude DNA repair defects. Thus, the chosen conditions are truly representative for analysis of PID-associated cancer frequency. A recently published series of studies of patients with primary immune deficiency, which included large cohorts and a comparison group, from either Australia (6), the Netherlands (7), or the USA (4), indicate that PID causes a significant increased risk of all cancers of 1.6-, 2.3-, or 1.42-fold, respectively, compared to the general population. This is much lower than the approximately 10,000-fold (45) increase estimated in past reports.

### Cancer Distribution in PIDs

Despite this global increased risk, the PID-cancer profile observed in the two largest studies, together including 4,790 patients, contained strikingly few cancer types. The Australian patients presented an excess of non-Hodgkin lymphoma (SIR 8.82), leukemia (SIR 5.36), and stomach cancer (SIR 6.10). Tumors were predominantly associated with antibody deficiencies (6). In the US study, the authors observed a 10-fold or 8.340-fold excess of lymphoma for men and women, respectively, but no increase for the most frequent common solid malignancies: prostate, lung, breast, and colon cancer (4).

A closer look at cancers observed in the nine selected conditions using epidemiological studies as well as institutional experience, series, and case reports allows for more precise tumor profiling. There is a global tumor profile for the nine conditions, and concurrently, a tumor profile for each condition. An excess of lymphoma occurred in the most diverse set of conditions, associating with five PIDs; however, liver and biliary tract cancer only associated with one. The majority of over-represented cancers were associated with either two or three conditions. Each condition in turn was associated with a narrow, sometimes very narrow, spectrum of cancers. For example, severe congenital neutropenia associated only with leukemia (40), and X-linked agammaglobulinemia associated only with gastric and intestinal carcinoma (19).

On the other hand, some tumors were rare in some of these diseases. In selective IgA deficiency, deaths from lung cancer were more than twofold less frequent than in the general population (14). In the whole group of PIDs, the most frequent cancers in the USA—breast, lung, and colon cancer—had a lower incidence of 0.41, 0.48, and 0.19, respectively, for women; for men, colon and lung cancer incidences were 1 and 0.62, respectively, compared to the general population. Interestingly, these are the most frequent epithelial malignancies, suggesting an underrepresentation of these types of cancers in the largest available study conducted (4). In CVID, lung, breast, and colon cancer had a lower incidence compared to that of the general population (4). Thus, the incidence of most solid tumors in PIDs is not significantly higher than in the general population. This is well shown by these epidemiological studies which took into account the reduced life expectancy (due to infection-associated sequelae or autoimmunity), for instance in CIVD, Wiskott–Aldrich syndrome, X-linked agammaglobulinemia, and X-linked hyper IgM syndrome, which calculated age-adjusted cancer incidence (4) and adjusted evaluation for 5-year age group (6). Some PIDs, outside of the nine selected, are not associated with malignancies. In contrast, they appear to have global decreased cancer incidences. This is the case for X-linked chronic granulomatous disease where no cancer was registered among 326 patients (4). Globally, cancer distribution in PID does not seem to result from a unique general mechanism applying to every disease. This does not fit with a defective immune surveillance model. The cancer distribution rather suggests that different mechanisms are involved, leading to special and sometimes unique tumor profiles.

### Possible Mechanisms

The largest and most recent study of patients with PID-associated cancers points to a restricted role of the immune system in protection from cancer (4).

#### Lymphoma

Lymphoma is unquestionably the most over-represented malignancy in PID, with an approximate 8- to 13-fold increased risk compared to that of the general population (4, 6, 7). These are mainly B cell lymphoma occurring in extranodal sites, often in patients with preceding polyclonal lymphocyte infiltrations (11). As emphasized by Hauck et al., in PIDs, cancer (lymphoma) affects cells altered by the deficiency itself (lymphocytes) (46). This increase in lymphoma has been linked to recurrent and persistent viral and bacterial infections, which characterize a great majority of PIDs sometimes in an antigen-specific manner (32, 47, 48). The overstimulated defective lymphoid tissue leads to lymphoid hyperplasia observed in 50% of patients with CVID. They show lympho-adenopathies and splenomegaly (17), a state favoring lymphoma transformation.

#### Leukemia

An increased incidence of leukemia, presenting as abnormal bone marrow, is observed in some PIDs. In severe congenital neutropenia, patients experience bone marrow failure (40). The bone marrow of NK cell-deficient patients is characterized by multilineage dysplasia (44). In these conditions, hematopoietic lineages could be the target of chronic overstimulation to counterbalance the impaired production of white cells. Elevated intrinsic G-CSF observed in severe congenital neutropenia also increases the risk for acute myeloid leukemia and myelodysplastic syndrome (MDS), in addition to the treatment with G-CSF (38). Reviewing chronic myelocytic leukemia (CML) in immune deficiency, Gale and Opelz found no global excess of CML in patients with immune deficiency. They suggested that failure of immune suppression *per se* does not explain most cases of CML and conclude that immune surveillance does no contribute significantly toward preventing new cases of CML (49). The role of immune surveillance could as well be questioned for other PID-associated leukemias.

#### Digestive Tract Cancers

Stomach cancer is over-represented in epidemiological studies on malignancies in PID, especially in humoral defects such as CVID and selective IgA deficiency (4–6). In conditions with humoral defects, the impaired immune system permits recurring *Helicobacter pylori* gastric colonization, which leads to gastritis, and, for some patients, to severe atrophy and intestinal metaplasia which are two major risk factors for gastric adenocarcinoma (16, 17). An increased incidence of intestinal cancer has been found in patients with X-linked agammaglobulinemia. In this disease, a team observed inflammatory bowel diseases and infectious enteritis, which are risk factors for cancer, for 11.3% of patients (21). Patients with X-linked hyper-IgM syndrome who present frequent protracted diarrhea also develop more colon cancer (23). Additionally, patients with X-linked hyper-IgM syndrome are much more prone to hepatitis and cholangitis, mainly linked to persistent infection of *Cryptosporidium Parvum*, and are anticipated to develop cancer in organs altered by chronic inflammation and cirrhosis (22).

#### Viral Infections

Cancers related to oncogenic viral infections account for 12% of cancers in humans and an even higher percentage in patients with primary or acquired immune deficiencies (9, 50). Cancers of the genital, anal, and oropharyngeal regions and of the skin (50, 51) are more frequent in those PIDs that are vulnerable to human papilloma virus (HPV) infections, particularly patients with NK cell deficiency who develop an excess of cervix-uterine HPV-related dysplasia and cancer as well as oropharyngeal squamous cell carcinomas and skin carcinomas (44). Genital and oral HPV-related squamous cell carcinomas are also observed in patients with WHIM syndrome (35, 36). Epstein–Barr virus (EBV) infections cause lymphoproliferative diseases and lymphoma, which are also observed in WHIM syndrome (34, 37), and in cartilage–hair hypoplasia (32). Additionally, EBV causes EBV-smooth muscle tumors, which have been reported in liver, adrenals, and smooth muscle of patients with NK cell deficiencies (42, 44). Significantly, in PIDs there is an excess of malignancies stemming from organs targeted by viruses but no excess of cancers in other tissues.

The molecular pathogenesis of PIDs associated with malignancy cannot be developed for each clinical condition included in Table 1; this is beyond the scope of the current article. NK cell deficiency, for example, has been associated with mutations in five genes, *GATA2, MCM4, RTLLE1, IRF8*, and *FCGR3A* (51). At the same time, *GATA2* deficiency has been linked to four clinical syndromes: NK cell deficiency, but also monocytopenia and mycobacterial infection syndrome, familial MDS and Emberger syndrome (44). *GATA2* is a transcription factor highly expressed in immature hematopoietic cells. The gene is necessary for survival and renewal of hematopoietic cells. It is critical for genesis and function of hematopoietic stem cells and thus blood cell lineage (44). Myeloid malignancy in *GATA2* deficiency is related to differentiation arrest and in part to a novel function of the mutated gene. Currently, it remains unclear how germline *GATA2* loss-of-function mutations result in myeloid neoplasms (52). In this context the hypothesis of an overstimulation of myeloid cells could be suggested.

## Discussion

### The Cancer Distribution in Animal Models of Immune Deficiency Is Similar to That of PID Patients

As animal models are considered to support the concept of immune surveillance, it is interesting to compare the spontaneous tumor occurrence in immune deficient mice to that in PID patients. An early study showed no difference in the incidence of spontaneous lung adenoma between athymic-nude mice, which are deficient for T cells, and immunocompetent mice (53). More recent works using immunodeficient mouse strains with defects in performin, interferon gamma, recombination activating gene (*Rag2*), signal transducer, and activator of transcription 1 (*Stat1*), and other genes reported an increased cancer incidence; however, the distribution of cancer resembled that observed in humans with PID. In a review of 11 strains, eight showed an excess of lymphoma and one an excess of plasmacytoma, either alone (6) or associated with other malignancies (3). Only two strains showed an excess of carcinomas and no excess of lymphoma (2). One team who studied spontaneous tumors in mice lacking the tumor necrosis factor (TNF)-apoptosis-inducing ligand (TRAIL) concluded that TRAIL-R did not protect against mammary cancers or against colon cancers, but did protect against lymphoid malignancies, which affected more than 25% of the deficient mice (54). An ongoing process of immune activation through IL6 upregulation has been proposed to explain plasma cell hyperplasia followed by plasmacytoma in mice lacking the interleukin-12 receptor Beta2 (55).

The literature describing mouse models of immune deficiency report increased incidence of lymphoma, while carcinomas are rare, as seen in human PID (see above). Notably, for the two mouse strains with only carcinomas, the tumor distribution is quite narrow. Mice lacking *Rag2* developed mainly intestinal adenomas and colon carcinomas (56), which usually occur following intestinal infection (57). Mice lacking both *Rag2* and *Stat1* developed an excess of colon cancer and breast carcinomas (56). As *STAT1* is involved in breast cancer pathways (58), the increase in breast cancer is likely due to STAT1’s role in tumorigenesis instead of immune surveillance. Similarly, a mouse model with a deficiency of granulocyte-macrophage colony stimulating factor exhibited an excess of both lymphoma and solid tumors. Interestingly, mice on antimicrobial therapy developed neither lymphoma nor solid malignancies compared to non-treated mice (*p* < 0.001). As the treated mice showed a marked reduction of both chronic infection and cancers, the authors proposed a role for chronic infection in the onset of malignancies (59). Thus, carcinomas observed in immune-deficient mouse strains are likely the consequence of specific processes affected by the deficient gene, for example, tissue damage secondary to unresolved inflammation, instead of a general decrease in immune surveillance, which should cause an excess of many types of carcinomas.

### Additional Observations Challenging Immune Surveillance

Other clinical observations indicate that immune defects increase the incidence of lymphoma instead of all cancers and cast doubt on the protective role of the immune system in cancer. First, the excess of lymphoma (but not other cancers) observed in Nijmegen breakage syndrome is associated with chronic stimulation of defective lymphocytes (60). In fact, only conditions with a chromosome breakage-DNA repair defect presenting with an immune deficiency, i.e., ataxia telangiectasia, Nijmegen breakage syndrome, and Bloom syndrome, develop an excess of lymphoma. In contrast, conditions with a chromosome breakage-DNA repair defect without immune deficiency, such as xeroderma pigmentosum, Fanconi anemia, Werner syndrome, and Rothmund–Thomson syndrome, develop other types of cancers. Second, although malignant melanoma, renal cell carcinoma, and non-small cell lung carcinoma have historically been considered as proof of immune therapy principles, these two cancers are not found more frequently in PIDs than in the general population (46). This is particularly clear in the nine conditions presented in Table 1. Third, although the immune system is weak and immature in neonates and young infants (61), there is no increased incidence of cancer at this period (62). In fact, a notable number of various neonatal cancers, some very aggressive, e.g., neuroblastoma, leukemia, and pontine glioma, may undergo spontaneous regression (62–64). Furthermore, in neonates, a cure for sarcoma is possible despite incomplete tumor resection (65). For this reason, the neonatal period was dubbed, three decades ago, the “oncogenic grace period” (66). Fourth, although people with Down syndrome have mildly to moderately weakened immune systems favoring pneumonias and opportunistic infections (67), adults with Down syndrome experience half the burden of solid tumors observed in the general population, notably showing a reduced frequency in the most common cancers: breast, prostate, and lung (68, 69).

### Is Cancer a Foreign Organism?

As the immune system’s role is to identify and destroy foreign organisms, bacteria, viruses, and parasites, the concept of cancer immune surveillance presupposes that cancer tissues are foreign (non-self) to the body of the patient (self). In this theoretical framework, cancer would be a foreign biological process characterized by genetic modifications. However, it is a genetic modification (gene or chromosome mutation) sufficient to consider a tissue foreign to the body? We should then consider that a person who has a constitutional genetic mosaicism as partly composed of “non-self” tissues. For instance, a woman with mosaic Turner syndrome with 20% 46, XX cells and 80% 45, X0 could be considered to be composed of 80% foreign tissue. A genetic point of view is really debatable for considering that a mutated tissue is foreign to the body. Even if they are genetically modified and even if they become uncontrolled by the integrating and coordinating system of the body, cancer tissues remain composed of body cells.

If cancers are recognized as foreign structures, the body would naturally reject them as it does for bacteria, viruses, parasites, and non-compatible grafts. Foreign tissues induce antibody production by the immune system, but tumor-specific antigens are not common in human tumors. Nonetheless, mouse models show that antigen expression is important for the process of recognizing and eliminating cancer cells (70). However, tumor reactive antibodies mirror tissue damage and reflect a response to tissue necrosis, rather than targeting a specific cell type (71). The observation that cancer cells, excepting those of virus-induced malignancies (70, 71), do not usually harbor strong specific antigens which suggests that cancers are not considered foreign by the body. Cancer could as well be seen as a diseased tissue. Some researchers propose that cancers can be considered as abnormal organs that develop in a manner similar to that of normal organs (72). Moreover, specific immune factors, such as a given major cytokine-like interferon gamma, or major immune effector cells, such as CD4 T lymphocytes, have both pro- and anti-tumoral effects (73, 74). These and other puzzling observations (75) suggest that the immune system has a complex reaction to tumor cells and does not always and systematically protect against malignancies.

Approaching cancer as a foreign organism implies that the main tumor and any tumor cells must be destroyed to cure the patient, similar to treatments aimed at killing bacteria, viruses, and parasites. This point of view seems a legacy of concepts and paradigms developed during the nineteenth and twentieth centuries with the progress of microbial medicine (76). We should not remain constrained by a framework that has been very effective for microbiology but narrows our understanding of oncogenesis. It is time to open our minds to other currently neglected approaches. We may question how best to fight against a disease composed of our own cells, which have escaped bodily control and gone awry. We may also question current cancer treatments that destroy normal cells likely because cancer is not so different from normal tissue.

Cancer could also be considered as a developmental disease, leading us to search for the processes that protect against malignancy during the embryonic and neonatal periods. The observation that embryonic carcinoma cells can incorporate into a normal blastocyst and produce a normal mouse composed of a mixture of normal cells (the host) and normalized cancer cells (the incorporated teratocarcinoma) (77) and that cancer cells in the presence of normal tissue may reverse to a normal phenotype (78) enlarges our perspective on research for cancer control and treatment. While the present study does not discuss the effectiveness of immune therapy and the progresses in treatment (79), we suggest that immune surveillance is probably not, in natural conditions, as effective as expected.

This review has some limitations, since only nine conditions were included. However, these conditions account for more than half of cancers reported in the largest epidemiological study on cancer in PIDs (4). Epidemiological studies are high-quality works, where the biological diagnosis is difficult to challenge. Isolated case reports and series are well documented, published in high-quality journals. Thus, repetitive errors on tumor diagnosis are unlikely.

## Conclusion

From the presented data we may draw the following conclusions: (1) The increased risk in frequency of cancer in PIDs is moderate, nearly twofold, much less than formally estimated. (2) The spectrum of cancer in PIDs is narrow. (3) Over-represented malignancies are mainly lymphoma, digestive tract tumors, and cutaneous-mucus carcinoma, which can be explained by an overstimulation of lymphocytes, chronic digestive infection, and cutaneous-mucus viral infections. (4) Animal models of immune deficiency develop a similar spectrum of malignancies as observed in human. Thus, since a defect of immune surveillance should theoretically favor all types of cancers, the data raise the hypothesis that immune surveillance does not play a major role in the increase of cancer in PIDs. While analyzing proposed mechanisms of oncogenesis and unmasking discrepancies with the immune surveillance model, three aspects of oncogenesis were emphasized, the overstimulation of a tissue leading to cancer, the role of tissue inflammation and fibrosis, and the possibility of cancer control by the body as it is observed during the neonatal period.

From an immediate point of view, it is disappointing to find that the role of the immune system is not, in a typical condition (i.e., non therapeutic conditions, particularly when excluding CAR T-cell therapy and immune checkpoint therapy), as important as expected. From another point of view, it is very exciting news implying that each PID with its specific excess or decrease of cancers has something to tell us about precise aspects of carcinogenesis. It is thus necessary to precisely identify the cancer types that occur and to articulate these data to functional and tissue alterations linked to the disease. These all provide many pathways for understanding cancer under precise conditions and possibly to fight against neoplasia.

## Author Contributions

The author confirms being the sole contributor of this work and approved it for publication.

## Conflict of Interest Statement

The author declares that the research was conducted in the absence of any commercial or financial relationships that could be construed as a potential conflict of interest.
